# Rheotaxially Grown and Vacuum Oxidized SnO_x_ Nanolayers for NO_2_ Sensing Characteristics at ppb Level and Room Temperature

**DOI:** 10.3390/s20051323

**Published:** 2020-02-28

**Authors:** Barbara Lyson-Sypien, Monika Kwoka

**Affiliations:** Department of Cybernetics, Nanotechnology and Data Processing, Faculty of Automatic Control, Electronics and Computer Science, Silesian University of Technology, 44-100 Gliwice, Poland; Monika.Kwoka@polsl.pl

**Keywords:** NO_2_ surface photovoltage gas sensor, RGVO SnO_x_ nanolayers, room temperature detection limit at ppb level

## Abstract

This work presents, for the very first time, very promising nitrogen dioxide (NO_2_) sensing characteristics of SnO_x_ nanolayers obtained by the innovative and unique rheotaxial growth and vacuum oxidation (RGVO) processing technique. The NO_2_ gas sensing experiments were performed using the novel surface photovoltage gas sensing device. The measured detection limit at room temperature (RT) is as low as 10 ppb NO_2_ in synthetic air, whereas the detection limit calculated on the basis of signal to noise ratio is around 6 ppb NO_2_. For the complementary study of surface chemistry of RGVO SnO_x_ nanolayers, including nonstoichiometry, presence of carbon contamination and surface bondings, the X-ray photoelectron spectroscopy (XPS) method was applied. The SnO_x_ RGVO samples reveal nonstoichiometry because the relative concentration [O]/[Sn] equals 0.94 for the as deposited sample and increases upon subsequent air exposure and NO_2_ sensing. Moreover, carbon contamination has been recognized after exposing the RGVO SnO_x_ nanolayers to the air and during the NO_2_ detection.

## 1. Introduction

Nitrogen dioxide NO_2_ can be considered as one of the main pollutants of the environment induced by industrial development in modern society. It can be harmful to living organisms not only by breathing in its vapors leading directly to serious illnesses of the airways, especially dangerous in the case of people suffering from asthma [1,2,3], but also by its indirect destructive impact on environment, including among others formation of acid rain and near ground level ozone [4,5]. In the case of NO_2_, exposure to a concentration larger than 1 ppm can lead to serious illnesses of the human respiratory system or aggravation of existing afflictions such as, among others, bronchitis, emphysema and lung insufficiency as well as worsen the medical condition of the circulatory system [6]. 

Having these dramatic consequences in mind, the issue of monitoring NO_2_ concentration, especially in urban areas, has recently became highly important. According to the current regulations of the European Parliament on ambient air quality and cleaner air in Europe [7] the limit value of NO_2_ concentration for an exposure of no longer than 1 h not exceeded more than 18 times a calendar year is 200 µg/m^3^, whereas the limit value for NO_2_ concentration in the case of the constant exposure in the calendar year cannot exceed 40 µg/m^3^.

The issue of monitoring NO_2_ can also have diagnostic applications, since it appears that the increase of its level in exhaled breath of people having asthma foreshadows an asthma attack and can also be used to identify respiratory system infections [8,9]. 

The requirement of NO_2_ monitoring in all the areas of human safety is nowadays a hot topic which motivates the search for modern and reliable nitrogen dioxide sensing material with the ability to recognize selectively ppb level concentrations of NO_2_, which appears to be a highly important facility. The issue of currently commercially available gas sensors for the monitoring of volatile organic compounds, including NO_2_ detection, was nicely reviewed in [10,11]. Spinelle et al. [10] concludes that as far as one is concerned with resistive sensors, SnO_2_ is nowadays the most commonly used material which enables the monitoring of NO_2_ concentration at the ppb level. However, according to [10], additional heating is required for this sensor to operate. Sulczynski et al. [11] mentions an electrochemical sensor produced by Environmental Sensors Co. which enables the detection of the presence of NO_2_ with a resolution of 0.1 ppm. From this point of view, it appears that there is still a lot to be done in the field of searching for new materials for the construction of a commercial NO_2_ detector with the ability to recognize ppb concentrations at relatively low temperatures.

Within the variety of NO_2_ sensitive materials, still the most important and significant group are resistive detectors based on semiconducting metal oxides such as: SnO_2_, ZnO, WO_3_ and In_2_O_3_. However, the simultaneous improvement in the detection limit towards single ppb together with the operating temperature lowered down to room conditions cannot be achieved without difficulty for a single, unmodified compound. In the case of pure SnO_2_, thin films deposited by reactive magnetron RF sputtering, Sharma et al. [12] reported the detection limit of 1 ppm NO_2_ at 80 °C. For SnO_2_ nanowires the detection limit of 120 ppb NO_2_ has been achieved at 140 °C, whereas the theoretical limit is reported to be as low as 0.062 ppb NO_2_ [13]. The improvement in terms of lowering the operating temperature with the simultaneous ability to recognize ppb concentrations of NO_2_ was observed by Y. Li et al. [14] for SnO_2_ nanoflowers, for which 50 ppb NO_2_ was detected at room temperature. 

Nowadays the most common approach to the issue of lowering the detection limit at room temperature in the case of SnO_2_ consists in applying catalysts and forming heterostructures not only with other metal oxides such as ZnO [15,16], WO_3_ [17] and NiO [18], but also with graphene [19,20,21] and carbon nanotubes [22,23] being under thorough investigation. 

Among the NO_2_ gas sensors studied recently there are also promising organic semiconductor-based sensors [24] with the detection limit of 1 ppb at room temperature. However, these form a new class of gas detectors. Another group of gas sensors that are promising in terms of long term stable NO_2_ detection are amperometric sensors with ionic liquid electrolytes—reviewed in [25]—which enable to recognize NO_2_ at room temperature [26] with response time at the level of several seconds [25,27] and the detection limit in the range of 30–90 ppb at room temperature [27]. As it can be concluded, in general the effort is directed toward searching for new and sophisticated materials which demand high-tech, time and money consuming procedures. 

Our aim within this work is to concentrate on the determination of the NO_2_ sensing features of SnO_x_ nanolayers obtained by the innovative and unique rheotaxial growth and vacuum oxidation (RGVO) technique. From our previous study [28,29], it is concluded that RGVO SnO_x_ nanolayers are prospective excellent candidates for gas sensing due to an increased surface to volume ratio, decreased agglomeration of nanograins, reduced presence of undesired carbon contamination and controlled non-stoichiometry. 

This work presents—for the very first time—highly enhanced gas sensing properties of pure RGVO SnO_x_ nanolayers towards NO_2_ sensing in sub ppb region at room temperature. The gas detection principle applied within this study is based on the surface photovoltage effect (SPV), which consists in measuring the variation of the near surface region potential barrier upon illumination and NO_2_ adsorption/desorption processes. The SPV gas sensor was successfully applied for ZnO thin films as described in our previous papers [30,31]. It enables to perform effectively low concentration level gas recognition at room temperature, whereas in principle, for metal oxide based conductometric gas sensors, additional heating is required.

## 2. Materials and Methods

SnO_x_ nanolayers were obtained using the rheotaxial growth and vacuum oxidation (RGVO) technique, the technique being our unique modification of rheotaxial growth and thermal oxidation (RGTO) method [32], which was described recently in detail in [28,29]. The samples with the thickness of 20 nm, controlled with quartz microbalance, were deposited at the Si (100) substrate by evaporation of Sn from the ceramic source under vacuum conditions related to 10^−4^ mbar of oxygen partial pressure. Additionally, these nanolayers were oxidized in situ at 400 °C with an oxygen partial pressure of 10^−2^ mbar for 2 h in order to increase their stoichiometry.

X-ray photoelectron spectroscopy (XPS) measurements were performed using a SPECS XPS spectrometer operating with Al Kα lamp (XR-50 source) and PHOIBOS-100 hemispherical analyzer. The XPS spectra of our RGVO SnO_x_ nanolayers registered in the various modes (survey, windows and lines) have been additionally calibrated with respect to reference binding energies (BE) using both the XPS Au4f peak at 84.5 eV, as well as the XPS C1s peak at 284.5 eV of residual C contamination, being always at the surface of all our samples under investigation.

The gas sensing experiments of our RGVO SnO_x_ nanolayers towards NO_2_ were performed using a novel type surface photovoltage gas sensor device operated at room temperature and described in detail in [30,31]. All the gas sensing measurements were performed with the total gas flow rate of 50 mL/min with the relative NO_2_ gas concentration in the synthetic air ranging from 10–500 ppb.

## 3. Results and Discussion

**Figure 1:**
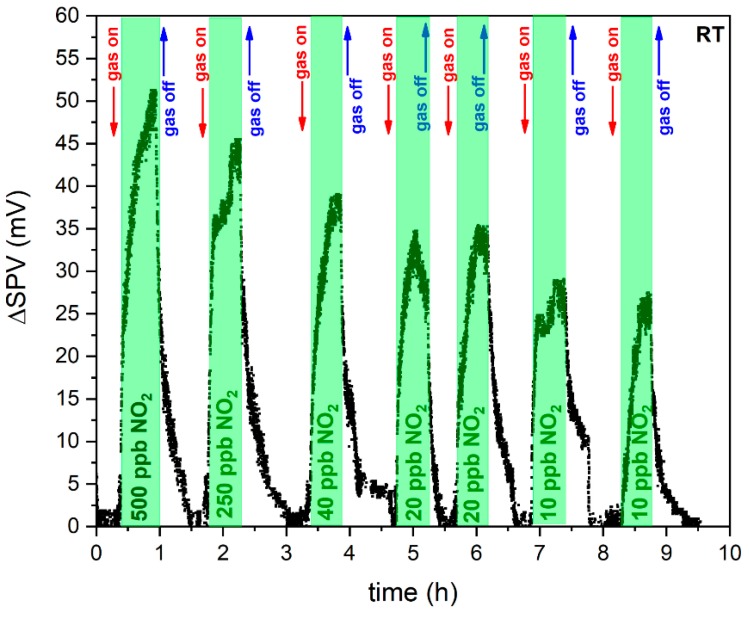
The variation of amplitude of surface photovoltage (SPV) signal for rheotaxial growth and vacuum oxidation (RGVO) SnO_x_ nanolayers after exposure to sequential relative concentration of 500, 250, 40, 20 and 10 ppb NO_2_ in synthetic air; areas depicted in green indicate the time interval when the step change of NO_2_ concentration appeared; the measurements were performed at room temperature (RT).

Figure 1 demonstrates the variation of amplitude of the surface photovoltage (SPV) signal of the RGVO SnO_x_ nanolayers after exposure to sequential relative concentration of NO_2_ in synthetic air in the range of 10–500 ppb. 

As it can be concluded, exposing the SnO_x_ RGVO nanolayer to NO_2_ induces a significant increase in the SPV value. As the gas flow of NO_2_ drops to zero, the baseline of the SPV signal is recovered. The time of response, *t_resp_*, defined as the period required to reach 90% of the final signal change, is in the order of a dozen minutes within the whole NO_2_ concentration range, for example in the case of the interaction with 500 ppb NO_2_ it equals (14 ± 2) min, 250 ppb: (15 ± 2) min, 40 ppb: (14 ± 2) min. The time of recovery is still rather long. However, it can be improved in the future by applying additional procedures to accelerate surface regeneration, such as infra-red illumination.

**Figure 2:**
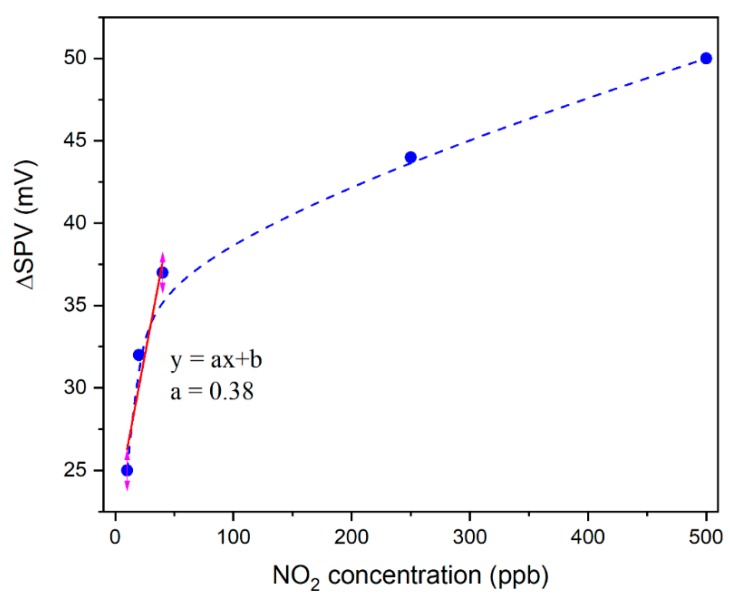
The variation of amplitude of surface photovoltage (SPV) signal corresponding to the gas sensor response for RGVO SnO_x_ nanolayers as a function of the relative concentration of NO_2_ in the synthetic air in the range: 10–500 ppb; the dashed line is a guide to notice; the linear function used for the calculations of the detection limit.

In the case of the exposure to 20 and 10 ppb NO_2_ (see Figure 1), the measurement was repeated in order to examine the short-time stability of the sensor response, which appears to present a satisfyingly good level, as the variation of amplitude of the SPV gas sensor signal for both 20 and 10 ppb NO_2_ obtained in the second step reaches the same value as the corresponding measurement performed previously. Moreover, as one can see, the variation of amplitude of the SPV gas sensor signal decreases as the RGVO SnO_x_ sample faces lower concentrations of NO_2_ (see Figure 2).

As it can be seen in Figure 1 and Figure 2 the detection limit for the RGVO SnO_x_ nanolayer is lower than 10 ppb NO_2_. In order to discuss theoretically the smallest amount of NO_2_ that could be detected, the signal to noise ratio was taken into account according to the International Union of Pure and Applied Chemistry recommendations [33], which specify that for reliable measurement the signal to noise has to be larger than 3. The procedure applied within this study, previously proposed by Li et al. [34] and successfully applied in literature [21,35,36,37] is given by Equation (1):

(1) $$DL=3\frac{rms_{noise}}{slope}=3\frac{\sqrt{\frac{\sum {\left(y_{i}-y\right)}^{2}}{N}}}{slope}$$

where *rms_noise_* denotes root mean square deviation between experimental data within baseline region, *y_i_*, and values fitted using polynomial function, *y*; *slope* corresponds to *a* coefficient in linear function: y=ax+b used for fitting the sensor response, Δ*SPV*, as a function of the gas concentration (see Figure 2); whereas, *N* denotes the number of data points taken into account for fitting—in this work *N* = 10.

On the basis of the procedure described above, it appears that the theoretical detection limit for NO_2_ recognition in the case of a SnO_x_ RGVO nanostructure is around 6 ppb. Undoubtedly, this result is unique for pure SnO_x_ sensing material working at room temperature.

**Figure 3:**
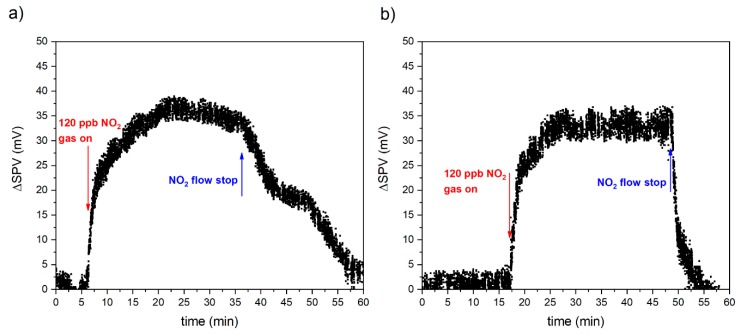
The variation of amplitude of surface photovoltage (SPV) for RGVO SnO_x_ nanolayers after exposure to 120 ppb NO_2_. The plot presented in (**a**) corresponds to the initial measurements, whereas (**b**) is related to the experiments repeated after 6 days.

In order to examine long term stability of RGVO SnO_x_ nanolayers, some of the gas sensing experiments were repeated. Figure 3 presented below demonstrates the response towards 120 ppb of NO_2_ obtained initially and after six days. As it can be concluded, in the case of 120 ppb NO_2_ the variation of amplitude of surface photovoltage (SPV) reaches a value in the range of 32–35 mV, which means that in both cases the sensor response is stable and repeatable. However, the recovery is noticeably faster for the measurement repeated after 6 days. This can be attributed to the real life, dynamic conditions that can affect the RGVO SnO_x_ nanolayers’ recovery. In the case of very low concentrations of NO_2_ in the ppb range, the sensor becomes significantly sensitive to the surrounding temperature and humidity. From this point of view, as it was mentioned above, one can consider applying some additional procedures, e.g., illuminating with IR radiation in order to speed up regeneration.

The issue of surface reactions in the case of SnO_x_ based gas-sensing material interacting with reducing and oxidizing gases together with the theoretical description linking the measured characteristics with the change in electron work function have been discussed in detail in [38,39,40,41].

The main limitation of work function variation defined as the contact potential difference, CPD, (measured using mainly the Kelvin probe approach) for the potential gas sensing application is its relatively poor sensitivity, related to the observed low values of the signal to noise ratio, as reviewed by Korotcenkov et al. [42]. This can be improved using the surface photovoltage effect SPV [43] which consists in measuring the variation of surface potential Δ*V_S_*, upon illumination at the well-defined constant radiation intensity *Io*, related to the charge redistribution appearing after photon induced electron-hole pair generation. The variation of SPV can be defined as:

Δ*SPV* ~ Δ*V_s_* ~ *I_o_* ⋅*V_s (after illumination)_* – *V_s (in dark)_* (2)

In our case the gas sensing mechanism is governed by the separation of charge carriers based on significant differences in their mobilities, which lead to the relatively large variation of electric potential within the space charge layer (SCL) observed finally as the variation of surface potential Δ*V_S_*. The fundamentals of the SPV technique were also briefly mentioned in our previous papers [30,31].

From our experience it appears that the observed Δ*SPV* values can even be in the range of hundreds mV. The variation of Δ*SPV* can be easily interpreted on the basis of the interaction of gas molecules with the surface of our gas sensing material (RGVO SnO_x_). It is commonly known that at low temperatures (below 150 °C) oxygen ions adsorb at the surface of metal oxide semiconductors in a form of O_2_^−^ [44] according to the equation:

(3) $$O_{2\left(\mathrm{gas}\right)}+e^{-}\to O_{2\left(\mathrm{ads}\right)}^{-}$$

leading in the case of SnO_2_ to the upward band bending due to the electrons’ trapping and depletion layer’s formation. 

In addition to the above, it is also generally accepted that oxidizing gases like NO_2_ adsorb at the surface of metal oxide semiconductor materials in an ionic form:

(4) $${\mathrm{NO}}_{2\left(\mathrm{gas}\right)}+e^{-}\to {\mathrm{NO}}_{2\left(\mathrm{ads}\right)}^{-}$$

according to Cho et al. [45] adsorption of NO_2_ competes with that of oxygen, which is given by Equation (3).

Both O_2_^−^ as well as NO_2_^−^ presence leads to the final surface charge density. The adsorption of NO_2_^−^ which is considerably stronger than that of O_2_^−^, according to [45], results in the further increase of the surface potential barrier *qV_S_*. 

In the case of semiconductors, the work function *Φ* is given as:

(5) $$\Phi ={\left(E_{C}-E_{F}\right)}_{b}+qV_{S}+\chi$$

where *(E_C_ – E_F_)_b_* denotes the difference between the energy of conduction band and the Fermi level in the bulk, *χ* is an electron affinity. In general, all the given components can change upon the interaction between the semiconductor surface and the gas phase. However, in our case both *(E_C_ – E_F_)_b_* as well as the *χ* parameters in Equation (5) can be treated as constant, as no bulk changes take place. What is crucial is that in our case the gas sensing mechanism is governed by significant changes in Δ*V_S_* promoted by the illumination, as described above.

**Figure 4:**
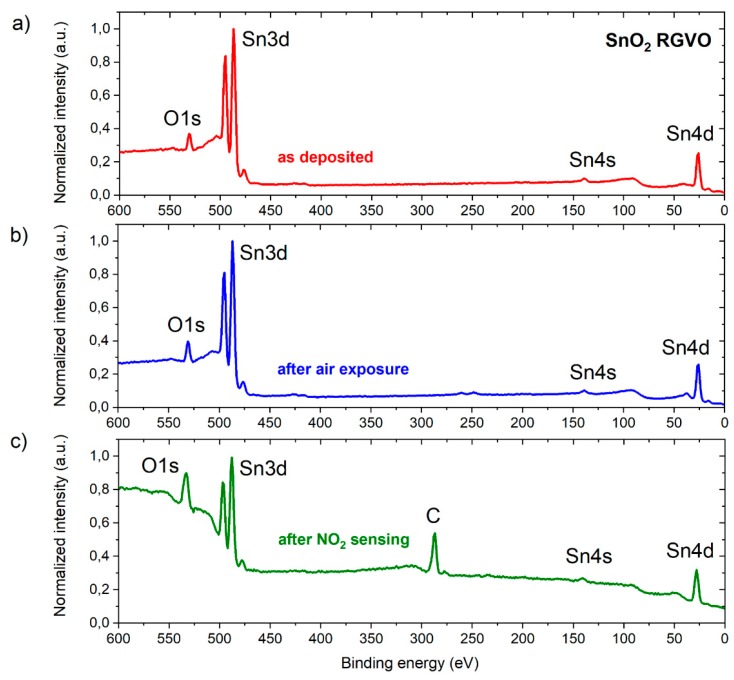
X-ray photoelectron spectroscopy (XPS) survey spectra with main core level lines of RGVO SnO_x_ nanolayers (**a**) as deposited; (**b**) after air exposure; (**c**) after NO_2_ sensing experiments.

In order to study surface chemical properties of RGVO SnO_x_ nanolayers, crucial for their gas sensing characteristics, X-ray photoelectron spectroscopy was applied. Figure 4 presents the XPS survey spectra for the as deposited sample, after air exposure as well as after subsequent NO_2_ sensing experiments.

**Figure 5:**
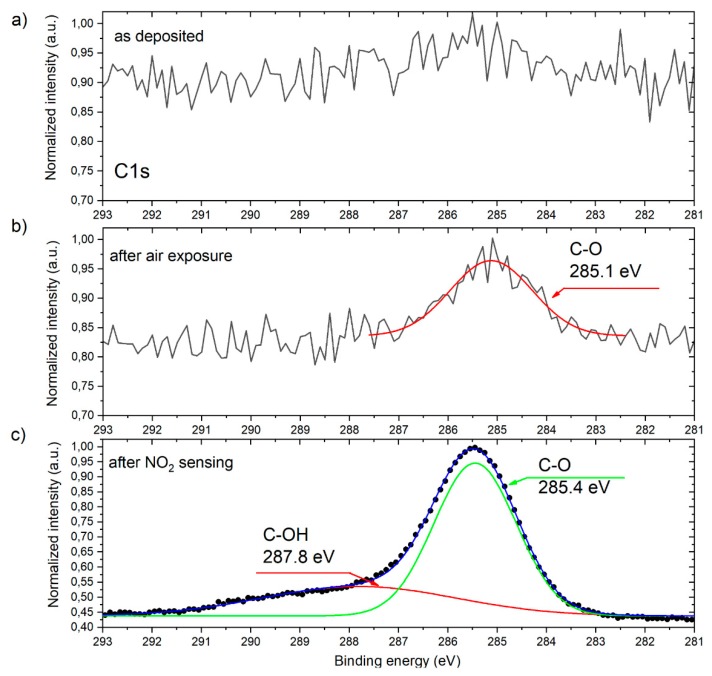
C1s spectral window for RGVO SnO_x_ nanolayers: (**a**) as deposited; (**b**) after air exposure; (**c**) after NO_2_ sensing experiments.

As can be clearly seen, the contribution from Sn and O is observed for the pristine and for both the air as well as NO_2_ exposed nanolayer. In the case of the sample which underwent NO_2_ detection, an evident carbon presence at the surface can be observed. Having in mind that carbon undesired contamination is crucial for subsequent gas sensing characteristics, the detailed XPS analysis of C1s spectral windows was applied (see Figure 5). As it can be concluded on the basis of Figure 5a, the RGVO SnO_x_ nanolayers elaboration procedure applied within this work does not trigger unwanted carbon contamination, as on the basis of the signal to noise ratio the contribution from C1s in this case is not observed. This fact undoubtedly can be interpreted as a great advantage of rheotaxial growth and the vacuum oxidation method. In the case of the RGVO SnO_x_ nanolayer which underwent air exposure (Figure 5b), a small contribution of carbon on the surface can be recognized and attributed to CO and CO_2_ adsorbed from the surrounding atmosphere [46,47]. For the sample after NO_2_ gas sensing experiments (Figure 5c) the amount of C increases. The deconvolution procedure of C1s spectral line shows that carbon present on the surface in this case comes from CO and CO_2_ (C-O component) as well as hydroxyl groups originating from dissociated water vapor [46,47].

In the second step for the more precise and quantitative analysis of XPS results, O1s – Sn3d as well as C1s spectral windows were used in order to calculate the relative concentration of the main components: [O]/[Sn] and [C]/[Sn], based on the atomic sensitivity factor (ASF) approach [48] and the procedure described in detail in our previous papers [49,50]. The results of this analysis are given in Table 1. As can be seen, the relative concentration [C]/[Sn] for the air exposed sample is as low as 0.08, whereas in the case of SnO_x_ it equals 3.96 after NO_2_ sensing. Perhaps this is related to the fact that small NO_2_ molecules promote hydroxyl group adsorption at the surface of our samples.

Moreover, on the basis of the results depicted in Table 1, one can consider the stoichiometry of the SnO_x_ RGVO nanolayers. It appears that the pristine sample is under stoichiometric with [O]/[Sn] equal to 0.94. After air exposure the [O]/[Sn] ratio increases to 1.09 as a result of additional oxidation which involves atmospheric oxygen. Furthermore, the interaction with nitrogen dioxide leads to the subsequent increase in the relative concentration of [O]/[Sn] which appears to be at the level of 1.37. The increase in the amount of oxygen upon interaction with NO_2_ can be attributed to the hydroxyl groups adsorption at the surface of our samples. However, still for all the samples, based on the relative concentration [O]/[Sn] values, under stoichiometry is observed. 

**Figure 6:**
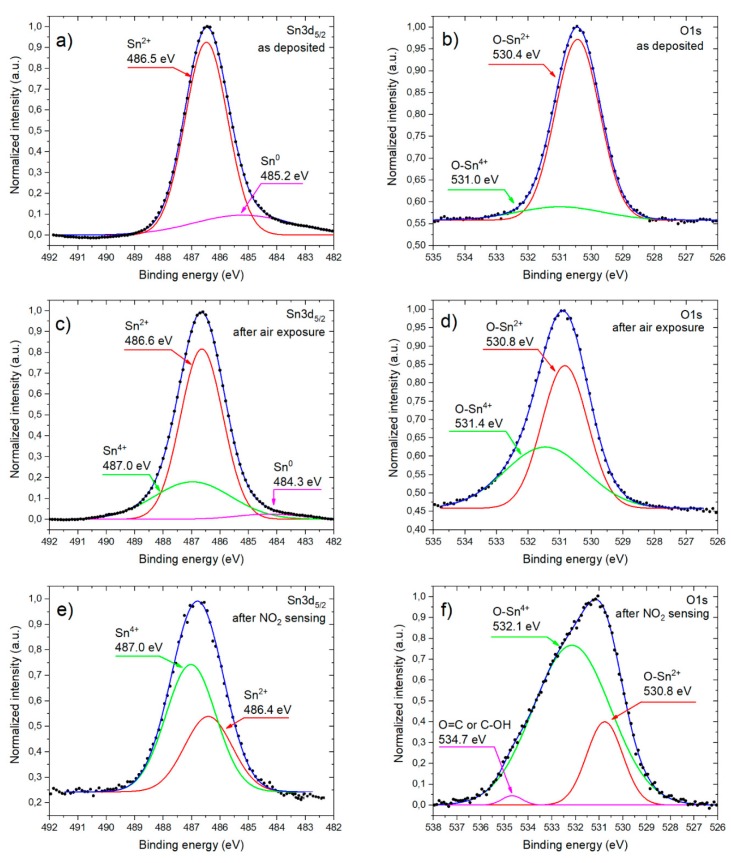
The decomposed O1s and Sn3d_5/2_ core lines of RGVO SnO_x_ nanolayer for the sample: as deposited (**a**,**b**); after air exposure (**c**,**d**); after NO_2_ exposure (**e**,**f**).

In the subsequent step the XPS O1s and Sn3d_5/2_ spectral lines were decomposed as can be seen in Figure 6. In the case of the as deposited sample, the predominant contribution of Sn^2+^ is observed both for the Sn3d_5/2_ as well as O1s decomposed lines (see Figure 6a,b). For O1s core line it appears that the two components related to O-Sn^2+^ (at 530.4 eV) and O-Sn^4+^ (at 531.0 eV) can be recognized. As far as the Sn3d_5/2_ line is discussed, one can conclude that the pristine sample contains also a small amount of metallic tin Sn^0^.

Exposing the RGVO SnO_x_ nanolayers to the air leads to some modifications in the chemical properties of their surface, being still a mixture of SnO_2_ and SnO as the contribution from the latter one decreases. Based on the deconvolution of Sn3d_5/2_ line (see Figure 6c) the contribution of Sn^2+^ (at 486.6 eV), Sn^4+^ (at 487.0 eV) and Sn^0^ (at 484.3 eV) can be recognized. As for the deconvolution of O1s (see Figure 6d), the two constituents are observed, i.e., O-Sn^2+^ (at 530.8 eV) and O-Sn^4+^ (at 531.4 eV). These results remain in good agreement with our previous paper [29].

In the case of the sample which underwent NO_2_ exposure, the O1s spectral line (Figure 6f) can be decomposed into three components attributed to O-Sn^2+^ (at 530.8 eV), O-Sn^4+^ (at 532.1 eV) and strong carbon contamination O=C or C-OH (at 534.7 eV). Decomposition of the Sn3d_5/2_ spectral line (Figure 6e) still reveals the impact of both Sn^2+^ (at 486.4 eV) and Sn^4+^ (at 487.0 eV). However, there is no contribution from metallic tin Sn^0^.

## 4. Conclusions

Within this study the novel RGVO SnO_x_ nanolayers were examined for possible NO_2_ detection at room temperature using the surface photovoltage effect. This gas sensing material is very promising in terms of gas detection because the experimental detection limit at room temperature is as low as 10 ppb NO_2_. In turn, the theoretical detection limit calculated on the basis of signal to noise ratio equals 6 ppb NO_2_. This means that our novel RGVO technique enables the obtaining of the promising gas sensor material, being a mixture of tin oxide SnO and tin dioxide SnO_2_, without undesired carbon contamination. This is very promising in terms of improving NO_2_ gas sensing characteristics. However, as it is generally, the C unwanted surface species usually obstruct the interaction between the semiconductor active surface and the gas under detection. In our experiments the carbon contamination appears only after exposing the sample to the air and more evidently after NO_2_ sensing as the relative [C]/[Sn] ratio equals to 0.08 and 3.96, respectively.

## Figures and Tables

**Table 1:** Relative concentrations of the main components of RGVO SnO_x_ nanolayers as deposited, after air exposure as well as and after NO_2_ sensing experiments.

SnO_x_ RGVO	[O]/[Sn]	[C]/[Sn]
as deposited	0.94	0.00
after air exposure	1.09	0.08
after NO_2_ sensing	1.37	3.96
